# Contrasting Codon Usage Patterns and Purifying Selection at the Mating Locus in Putatively Asexual *Alternaria* Fungal Species

**DOI:** 10.1371/journal.pone.0020083

**Published:** 2011-05-19

**Authors:** Jane E. Stewart, Masato Kawabe, Zaid Abdo, Tsutomu Arie, Tobin L. Peever

**Affiliations:** 1 Department of Plant Pathology, Washington State University, Pullman, Washington, United States of America; 2 Departments of Mathematics and Statistics and Initiative of Bioinformatics and Evolutionary Studies, University of Idaho, Moscow, Idaho, United States of America; 3 Institute of Symbiotic Science and Technology, Tokyo University of Agriculture and technology (TUAT), Fuchu, Tokyo, Japan; The Salk Institute, United States of America

## Abstract

Sexual reproduction in heterothallic ascomycete fungi is controlled by a single mating-type locus called *MAT1* with two alternate alleles or idiomorphs, *MAT1-1* and *MAT1-2*. These alleles lack sequence similarity and encode different transcriptional regulators. A large number of phytopathogenic fungi including *Alternaria* spp. are considered asexual, yet still carry expressed *MAT1* genes. The molecular evolution of *Alternaria MAT1* was explored using nucleotide diversity, nonsynonymous vs. synonymous substitution (*dn/ds*) ratios and codon usage statistics. Likelihood ratio tests of site-branch models failed to detect positive selection on *MAT1-1-1* or *MAT1-2-1*. Codon-site models demonstrated that both *MAT1-1-1* and *MAT1-2-1* are under purifying selection and significant differences in codon usage were observed between *MAT1-1-1* and *MAT1-2-1*. Mean GC content at the third position (GC3) and effective codon usage (ENC) were significantly different between *MAT1-1-1* and *MAT1-2-1* with values of 0.57 and 48 for *MAT1-1-1* and 0.62 and 46 for *MAT1-2-1*, respectively. In contrast, codon usage of *Pleospora spp.* (anamorph *Stemphylium*), a closely related Dothideomycete genus, was not significantly different between *MAT1-1-1* and *MAT1-2-1*. The purifying selection and biased codon usage detected at the *MAT1* locus in *Alternaria* spp. suggest a recent sexual past, cryptic sexual present and/or that *MAT1* plays important cellular role(s) in addition to mating.

## Introduction

Sexual reproduction in heterothallic ascomycete fungi is initiated when strains of opposite mating type interact, and this interaction is controlled by a single regulatory locus called *MAT1* [1]. The *MAT1* locus has two alternate alleles, *MAT1-1* and *MAT1-2*, which lack sequence similarity and have been termed idiomorphs [2]. All ascomycete *MAT1* idiomorphs encode proteins with confirmed or putative DNA binding motifs (e.g., *MAT1-1-1* = alpha box; *MAT1-2-1* = high mobility group [HMG] box), suggesting that *MAT1* genes encode transcriptional regulators which control the expression of additional genes required for sexual reproduction and possibly other cellular processes [3]. Little is known about the targets of these regulators in filamentous fungi [1], [4], however, these mating regulators have been well- studied in yeast [5]. *MAT1* loci are also found in putatively asexual ascomycete species [6]–[10]. It has also been demonstrated that genes at the *MAT1* locus are expressed in the asexual species, *Alternaria alternata*, and this locus was able to functionally complement a *MAT1*-null mutant in the closely related sexual species *Cochliobolus heterostrophus* [7].

A large number of phytopathogenic fungi are considered to be asexual (mitosporic) because they have no known teleomorph, yet still carry functional *MAT1* genes [6]–[7]. The role of *MAT1* genes in asexual fungi is unknown although most or all may be recombining through cryptic meiosis or a parasexual cycle [8]–[9], [11]. Direct observation of microbial reproduction in nature is difficult and most studies that have inferred recombination in putatively asexual fungi have relied on indirect inference using gametic disequilibrium or parsimony tree length comparisons among molecular markers [8]–[9], [11]–[12].

*Alternaria* is considered to be a largely asexual genus; however, *A. infectoria* has been connected to a *Lewia* teleomorph [13] and occupies a basal position in the phylogeny of the genus [8]. This suggests that most or all *Alternaria* species may have had sexual ancestors as has been suggested for other asexual ascomycetes [14]. A formal study to determine the mating system of *A. alternata* has not been conducted and we are currently unable to rule out the possibility that *Alternaria* genomes may regularly recombine through cryptic meiotic or parasexual processes. Results of Peever et al. [15] found two genetically distinct clusters of *A. alternata* infecting citrus within a small area (250 m^2^) of a single citrus grove. The strongly clonal population structure within each cluster was suggestive of asexual reproduction [15], although small sample sizes and lack of genetic linkage analyses of the markers precluded critical estimation of the mating system.

Molecular evolution analyses can be used to uncover patterns in codon usage to help reveal functionality, expression levels, and mechanisms of natural selection acting on a gene [16]–[17]. Codon usage has been shown to be highly biased [18], reflecting a balance among the forces of mutation, selection, and random genetic drift [19]. This bias appears to maximize the efficiency of translation [20], and differs among species due to changes in the complement of tRNAs or life history [21]–[23]. Comparing rates of synonymous and non-synonymous substitutions across a gene can provide powerful inferences about gene evolution [24]. Synonymous substitutions, codon changes that do not result in amino acid changes, are thought to be largely invisible to natural selection whereas non-synonymous substitution changes that lead to changes in amino acid may be under strong selective pressures. Purifying selection occurs when non-synonymous changes are suppressed and is thought to be a common force in maintaining gene function [25]. Several examples of fungal genes under purifying selection include ubiquitin, a protein that plays a major role in cellular stress response and protein degradation in eurkaryotes, was found to be conserved among 28 species of fungi, plants and animals [26], and the telomere-linked helicase gene family of *Magnaporthe oryzae* [25]. Positive selection, where non-synonymous changes are favored, has been identified in fungal genes related to defense-related genes and toxin protein genes [27]–[29] such as the phytotoxic protein-encoding genes (*NEP1* and *NEP2*) from *Botrytis* [30] and the host-specific toxin gene (*SnToxA*) from *Phaeosphaeria nodorum* [31].

The objective of this study was to infer the evolutionary processes acting on the mating type-locus, *MAT1*, in a filamentous fungus with no known sexual state. Sequence data from the *Alternaria MAT1* locus were used to estimate the direction and strength of selection acting on *MAT1* genes and to compare codon usage patterns to other genes in the *A. alternata* genome and *MAT1* genes in related species. Diversity, neutrality and codon usage patterns of *MAT1* of *Alternaria* were compared to *MAT1* of a closely related dothideomycete genus *Pleospora* (Anamorph *Stemphylium*) to test the hypothesis that *MAT1* in these closely related genera have similar signals of molecular evolution.

## Materials and Methods

### Fungal culture and DNA extraction

Twenty-one isolates of *Alternaria spp.* were used in this study (Table S1). *MAT1* sequence data from 16 closely related, heterothallic, and putatively sexually reproducing *Pleospora* spp. (Table S1) were downloaded from GenBank and TreeBase. These taxa were previously described as having typical heterothallic *MAT1* locus organization by Inderbitzin et al. [32] but were not verified as heterothallic in laboratory matings. For simplicity, these outgroup taxa are referred to using their anamorphic name, *Stemphylium*. Sequence data for 11 housekeeping genes in *A. alternata* and *A. brassicicola* were downloaded from GenBank and the DOE Joint Genome Institute (*Alternaria brassicicola* genome sequence) for comparison with *Alternaria MAT1* sequences to obtain baseline codon usage patterns for *Alternaria* spp.

For DNA extraction, fungi were cultivated in liquid 2YEG medium (2 g yeast extract, 10 g glucose per liter) for 1 week at room temperature on an orbital shaker at 150 rpm. Genomic DNA was extracted from powdered, lyophilized mycelium following the methods of Peever et al. [15], quantified using a spectrophotometer, and used as template for PCR. Isolates were maintained in long-term storage on sterilized filter paper at 4°C [15].

### PCR Amplification of *MAT1* sequences from *Alternaria* species

Mating type of each isolate was determined using mating type-specific PCR with primers designed to the *MAT1-1* (GenBank accession AB009451) and *MAT1-2* (GenBank accession AB009452) idiomorphs of the Japanese pear pathotype of *A. alternata* [7]. The full length *MAT1* gene (1.5 kb for *MAT1-1-1* or 2.4 kb for *MAT1-2-1*) was amplified from *A. alternata* using AAM1-11+AAM1-12 or AAM2-1+AAM2-2 (7) (Table 1), respectively. The full length *MAT1-1-1* gene was amplified from isolates SH-MIL-11s, SH-MIL-22s, SH-MIL-34s and SH-MIL-38s and full length *MAT1-2-1* was amplified in isolates SH-MIL-1s, SH-MIL-13s, SH-MIL-14s and SH-MIL-19s (Table S1). Twenty microliter PCR reaction mixtures contained 20 ng genomic DNA, 1×PCR buffer (New England Biolabs (NEB), Ipswich, MA), 4 nmol of each dNTP (NEB), 50 pmol primer, and 1 U of Taq polymerase (NEB). Cycling conditions consisted of denaturation at 94°C for 4 min; 44 cycles of 94°C for 1 min, 58 or 55°C for 30 sec, and 72°C for 2 min; final extension was at 72°C for 7 min depending on the optimal conditions for each primer set.

**Table 1:** Primers used to amplify *MAT1* locus from *Alternaria* spp.

Primer Name	Sequence (5′ to 3′)	Position	Description	References
AAM1-11	CATCATGATCATTGTTGT	252–269^1^	*A. alternata* full length *MAT1-1-1*	this study
AAM1-12	GCACACCTCAAGTGATCA	2650–2633^1^	*A. alternata* full length *MAT1-1-1*	this study
AAM2-1	TAGCGTTTGTCCGTACCGA	750–768^2^	*A. alternata* full length *MAT1-2-1*	this study
AAM2-2	GTAACGAGCATGAACATT	2211–2228^2^	*A. alternata* full length *MAT1-2-1*	this study
ASML-1	GGGTTGTTGTGGTCAAGGTT	144–163^1^	*Alternaria* spp. full length *MAT1-1-1* or *MAT1-2-1*	this study
ASMR-1	GTCATGATCAAGCAAGGGCA	2584–2565^1^	*Alternaria* spp. full length *MAT1-1-1* or *MAT1-2-1*	this study
AaM1-8	GGTCGTGAGTCGTGATCG	2257–2240^1^	*Alternaria* spp. full length *MAT1-1-1* or *MAT1-2-1*	this study
ASML-2	GGACGCATCGCAGATTGGAA	170–189^1^	*Alternaria* spp. full length *MAT1-1-1* or *MAT1-2-1* (nested PCR)	this study

1 nucleotide position based on GenBank accession AB009451(*MAT1-1-1*).

2 nucleotide position based on GenBank accession AB009452 (*MAT1-2-1*).

Several approaches were used to amplify the entire *MAT1* idiomorph from other *Alternaria* species because primers designed for *A. alternata* did not yield amplicons of the expected size for *A. brassicicola*, *A. brassicae* and *A. solani*. Full length *MAT1* genes (2.4 kb for *MAT1-1-1* or 2. 2 kb for *MAT1-2-1*) from *A. brassicicola* isolates 01-1c-s, 01-2a-s, 01-9c-s, 01-23a-s and 01-41a-s were amplified using primers ASML-1+ASMR-1 or AsM1-8+ASML-2, respectively (Table 1). Nested PCR was used to amplify *MAT1* from *A. brassicae* and *A. solani* isolates 01-8a, 01-8b, 21ss, 39ss, IdahoA and EGS 44-098 (Table S1). Primers ASML-1 and ASMR-1 amplified *MAT1* with a primary PCR reaction. This reaction yielded product containing several amplicons. PCR products were diluted 50-fold and were used as template for a second PCR reaction using primers ASML-2 and AaM1-8 to target a fragment containing full length *MAT1* gene only. Primary and secondary PCR conditions consisted of denaturation at 94°C for 4 min; 44 cycles of 94°C for 1 min, 58 or 55°C for 30 sec, and 72°C for 2 min; final extension was at 72°C for 7 min.

Amplified DNA fragments were sequenced directly on both strands following treatment with EXOSAP-IT (USB, Cleveland, OH) using the Big Dye terminator kit (Applied Biosystems, Foster City, CA). Sequence reads were performed on a PE Biosystems model 3700 automated DNA Sequencer by the Laboratory for Biotechnology and Bioinformatics at Washington State University, Pullman, WA. Sequences of *MAT* genes have been deposited under GenBank accession numbers GU735410–GU735428 (Table 1).

### Evolutionary analyses of *MAT1* genes in *Alternaria spp.* and closely related species

*MAT1-1-1* and *MAT1-2-1* ORFs from *A. alternata, A. brassicicola, A. brassicae, and A. solani* were predicted using two *A. alternata* reference isolates, 15A and O-276 carrying *MAT1-1-1* and *MAT1-2-1*, respectively [4]. Sequences were aligned manually and using DIALIGN [33]. Comparisons were made between *MAT1-1-1* and *MAT1-2-1* of *Alternaria* spp. and *MAT1* of putatively asexual *Alternaria* spp. and putatively sexual, heterothallic *Stemphylium* spp. (Table S1). Sequence diversity (*S*), the number of segregating sites, number of haplotypes (N_hap_) and haplotype diversity (H*_d_*) were estimated using DnaSP v4.5 [34]. Sequence diversity was also quantified using Watterson's *θ* parameter [35]. To determine if the patterns of polymorphisms and divergence within and among groups deviated from neutral evolution predictions [36], Tajima's D, D_T_ [37] and Fu and Li's D, D_FL_ [38] tests were performed. D_T_ compares the number of segregating sites to the average number of pairwise nucleotide differences. D_FL_ compares the number of recent (external branches) and ancestral (internal branches) mutations on a phylogenetic tree. Under a neutral evolution model, numbers of mutations on internal and external branches are expected to be equal. Increased number of external branch mutations indicates purifying selection whereas increased number of mutations on internal branches indicates balancing selection [39]. The significance of D_T_ and D_FL_ was tested using coalescent simulations where a neutral coalescent process was used to simulate 1,000 replicates with the number of segregating sites set to the observed data. When positive selection is acting, D_T_ tends to be positive, whereas D_T_ is negative in cases of purifying selection [40].

Signatures of purifying or positive selection acting on *MAT1* were tested at the codon level using codon-based likelihood analyses. A maximum likelihood implementation was used to fit codon substitution models to the data using the CODEML program within PAML v 4.2 [41]. Three random site models were used to describe the variation of ω ( = *d_N_/d_S_*) among codon sites within each *MAT1* gene. Random site models M0 (one ratio), M7 (beta) and M8 (beta&ω) [42]–[44] were used to describe the variation of ω (ω = d_N_/d_S_) among codon sites within each *MAT1* gene. M0 is the simplest model, assuming one ω for all codons in a dataset, which can be used to check parameter estimates in the other more complex models. M7 is a flexible null model in which a ω ratio for each codon is randomly selected from a beta distribution between 0 and 1. M8 adds one additional site class to M7 allowing for positive selection (ω>1). A test for positive selection was implemented using likelihood ratio tests (LRT) that compare models M7 and M8. The Bayes Empirical Bayes (BEB) approach [44] was used, within CODEML, to estimate ω using model M8 for each codon site for *MAT1-1-1* and *MAT1-2-1*. BEB also calculates the posterior probability (pp) that a codon site is from a positive-selection site class (ω>1), determining which codon sites are under positive selection, or from a purifying selection class (ω<0.25) [44]. Codon sites with ω>1 and ω<0.25 and pp values greater than 80% were considered to be under positive or purifying selection, respectively [44]. The tree file used as an input file for CODEML was produced using the parsimony search criterion in PAUP.

Codon bias, where certain codons are used preferentially, has been described in many organisms [45]–[46] and can give insight into translation efficiency and levels of protein expression [47]. Three measures of codon usage were used to estimate codon bias at *MAT1* of *Alternaria* spp. using CodonW 1.4.2 [48], the frequency of G+C at the third synonymous variable codon position (GC3), a measure of the effective number of different codons used in a gene (ENC) [49], and codon adaptation index (CAI) which is a univariate measure of synonymous codon usage used as an indicator of gene expressivity. CAI is the geometric mean of relative synonymous codon usage (RSCU) values, which is the observed frequency of a codon divided by the frequency expected under the assumption of equal usage of synonymous codons for an amino acid [50]. To obtain baseline codon usage patterns for *Alternaria* spp., 11 housekeeping genes from *Alternaria* spp. were compared (Table S1). GC3 and ENC were plotted and compared to ENC*, which is the null hypothesis that GC bias at the third position is solely due to mutation rather than selection [49], [51]. Genes lacking codon bias are expected to have an ENC score of 61, where all possible codons are used [49]. The reference species used for CAI analyses was *Saccharomyces cerevisae* [52]. Using this comparison, highly expressed genes that use the same codon set as *Saccharomyces cerevisae* would have a CAI value of 1. Lower values of CAI indicate smaller levels of expression [50]. The Mann-Whitney U test [53], implemented in R (v2.8.1) was used to determine if observed differences in ENC, GC3 and CAI between *MAT1-1-1*, *MAT1-2-1*, and housekeeping genes were significant.

## Results

### Evolution and patterns of purifying selection on *MAT1 of Alternaria*

Analyses of nucleotide variation between *Alternaria MAT1-1-1* (2.4 kb) and *MAT1-2-1* (2.2 kb) without regard to codon structure revealed no statistically significant differences in diversity (Table 2). *MAT1-1-1* and *MAT1-2-1* had similar diversity (within one standard deviation of each other) for variable sites (S) and nucleotide diversity (Watterson's *θ*). We failed to reject the null hypothesis of neutrality using Tajima's D (D_T_), which tests for neutrality using the average number of pairwise nucleotide differences. This indicated that nucleotide diversity at *MAT1* appeared not to deviate from neutrality when nucleotides across the entire gene were compared (Table 2). However, analyses of Fu and Li's D (D_FL_), which examines the distribution of mutations on internal or external branches, found that *Alternaria MAT1-1-1* was significantly greater than 1 (D_FL_ = 1.6, *P*<0.05), indicating that more mutations were found on internal branches, suggestive of positive selection.

**Table 2:** Genetic diversity and results of neutrality test of putatively asexual *Alternaria spp. MAT1* and closely related putatively heterothallic sexual *Stemphylium spp*.

	*MAT 1-1-1*			*MAT 1-2-1*		
Summary statistic	*Alternaria spp.*	*Stemphylium spp.*	All isolates	*Alternaria spp.*	*Stemphylium spp.*	All isolates
N	11	10	21	10	6	16
L	1173	1173	1173	1039	1039	1039
H	7	9	16	7	6	13
H*_d_*	0.927	0.978	0.976	0.933	1.00	0.97
S	267	301	599	202	181	483
*Π*	0.095	0.094	0.21	0.086	0.085	0.208
	0.078	0.093	0.146	0.069	0.076	0.143
D_T_	1.06	−0.39	0.73	0.98	−0.52	0.99
D_FL_	1.61^P^	−0.47	0.84	1.29	−0.64	1.24

### Signature of selection at *Alternaria MAT1*

**Figure 1:**
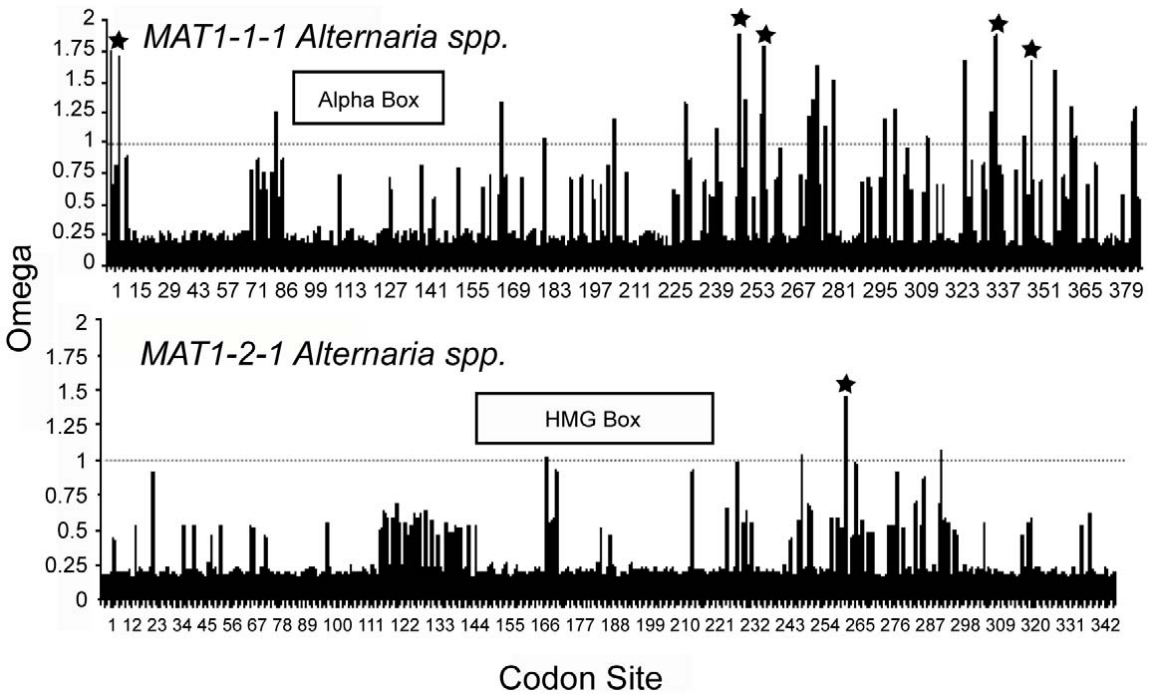
Non-synonymous to synonymous substitution ratio (ω) for *MAT1-1-1* (upper) and *MAT1-2-1* (lower) in *Alternaria* estimated in CODEML in PAML. The graph shows approximate posterior means of ω calculated as an average of ω's weighted by their posterior probabilities of the 11 site classes using in model 8a. The 11 ω ratios for *MAT1-1-1* are 0.08896, 0.10488, 0.11513, 0.12372, 0.13175, 0.13979, 0.14838, 0.15827, 0.17113, 0.19391, and ω_s_ = 1.46207. The 11 ω ratios for *MAT1-2-1* are 0.08585, 0.12609, 0.15474, 0.18009, 0.20463, 0.22990, 0.25745, 0.28968, 0.33196, 0.40659, and ω_s_ = 2.22313. Sites with low mean ω are inferred to be under purifying selection. Asterisks indicate sites with posterior probabilities more than 0.80 for ω_s_>1.

Using the site-branch model in PAML, we failed to reject the null hypothesis of neutrality using a likelihood ratio test comparing the M7 (null) and M8 (positive selection) site models across the entire genes of *MAT1-1-1* and *MAT1-2-1*. However, tests for selection using a codon-site model (BEB approach), which focuses on selection at individual codons, revealed an overall signal purifying selection acting on *Alternaria MAT1* with potential signatures of positive selection at few specific codon sites within *MAT1-1-1* and *MAT1-2-1* (pp greater than 80%). For *MAT1-1-1*, five of 387 codon sites had ω values greater than one with a pp over 80%, indicating possible positive selection. Two hundred and twenty five sites had a ω<0.25 at a pp over 90%, indicating purifying selection and of these 110 sites had a pp over 98% suggesting strong purifying selection (Figure 1). For *MAT1-2-1*, only one site had a ω over 1 with a pp greater than 80%, indicating possible positive selection. Of 342 codon sites, 124 had a ω<0.25 at a pp over 90% indicating purifying selection. When the pp stringency was decreased to 80% for *MAT1-2-1*, 256 of 342 sites were shown to be under purifying selection, indicating that 75% of the codons within *MAT1-2-1* are possibly under purifying selection. ω values for each of the conserved protein regions within each idiomorph, alpha box of *MAT1-1-1* (aa. 70–127) and HMG box of *MAT1-2-1* (aa 129–205) also provided evidence for purifying selection (Figure 1).

**Figure 2:**
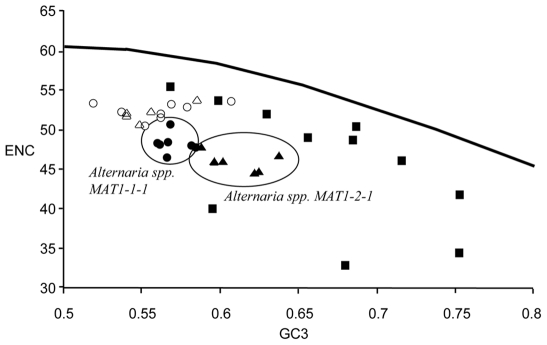
Effective number of codons (ENC) used in a gene plotted against the G+C content at the synonymously variable third position (GC3), for 21 *MAT1-1* genes, 16 *MAT1-2* genes, and 11 highly conserved genes (Table S1). Circles indicate *MAT1-1-1* gene of *Alternaria* (black), and *Stemphylium* (white). Triangles indicate *MAT1-2-1* genes of *Alternaria* (black) and *Stemphylium* (white). Squares indicate highly conserved genes of *Alternaria spp.* and *A. alternata* (black). The solid line is the expected ENC* curve, representing the null hypothesis that GC bias at the third position is solely due to mutation rather than selection.

Observed differences in effective codon usage (ENC), GC (GC3) content at the third position, and codon adaption index (CAI) between *Alternaria MAT1-1-1* and *MAT1-2-1* indicated divergent codon usage patterns. Significant differences for GC3 and ENC (*P*<0.001) were detected between *MAT1-1-1* and *MAT1-2-1* within *Alternaria*. Means for each were 0.57 and 48 for *MAT1-1-1* and 0.62 and 46 for *MAT1-2-1*. Overall, *MAT1-2-1* of *Alternaria* had larger GC3 values and lower ENC values, similar to housekeeping genes where no significant differences were observed (Figure 2). GC3 and ENC values plotted for all *MAT1* genes were significantly different from those of ENC*, indicating that selection was likely driving biased codon usage (Figure 2).

Comparisons of codon adaptation index (CAI) mean values of *Alternaria MAT1* and housekeeping genes indicated that *MAT1* had reduced levels of expression compared to housekeeping genes. Mean CAI values for *MAT1-1-1* and *MAT 1-2-1* were 0.107 and 0.095, respectively (Table 3). These values were lower than CAI values observed for *Alternaria* housekeeping genes which had a mean CAI of 0.151. *MAT1-2-1* had significantly smaller CAI values than *MAT1-1-1*, indicating lower expression. *MAT1-1-1* was not significantly different than housekeeping genes, though this was most likely the result of the high variance in CAI values of the housekeeping genes (Table 3). High variance within *Alternaria* housekeeping genes can be attributed to the diversity of genes incorporated in the analyses, which included an endoxylanase, exoglucanse, chitin synthase, kinase and G protein (Table 3).

**Table 3:** Mean codon adaptation index (CAI) values for *Alternaria MAT1* (Am1 and Am2), housekeeping genes (Ahk), and *Stemphylium MAT1* (Sm1 and Sm2).

Group	N^A^	Mean±STD	Pairwise comparisons				
			Am1	Am2	Ahk	Sm1	Sm2
**Am1**	11	0.107±0.004	-	*P* = 0.0005*	*P* = 0.262	*P = *0.001*	*P* = 0.116
**Am2**	10	0.095±0.005		-	*P* = 0.001*	*P* = 0.0002*	*P* = 0.001*
**Ahk**	11	0.151±0.069			-	*P* = 0.716	*P* = 0.925
**Sm1**	10	0.117±0.007				-	*P* = 0.080
**Sm2**	6	0.111±0.003					-

*Significant pairwise comparisons.

A Number of genes included in each group.

### Comparisons of nucleotide variation and codon usage at *MAT1* in *Alternaria* and *Stemphylium*

*Alternaria* and *Stemphylium MAT1* had similar numbers of polymorphic nucleotide sites and levels of diversity. All values were within one standard deviation of each other. Results examining nucleotide differences and diversity between *Stemphlium MAT1-1-1* and *MAT1-2-1* also yielded no significant differences results, as observed with *Alternaria*.

Codon usage patterns (ENC, GC3 and CAI) between *Alternaria* and *Stemphylium MAT1* were significantly different. *MAT1* of *Stemphylium* had significantly smaller GC3 (*P*<0.01), larger ENC (*P*<0.01), and higher CAI values (Table 3) compared to *Alternaria MAT1-2-1*. Comparisons of *Stemphylium MAT1* and *Alternaria MAT1-1-1* yielded no significant differences for GC3, but *Stemphlium MAT1* had significantly larger ENC values. Results of mean CAI value comparisons showed mixed results; *Stemphylium MAT1-1-1*, but not *MAT1-2-1*, CAI means were significantly larger than *Alternaria MAT1-1-1*.

Overall, codon usage between *Stemphylium MAT1-1-1* and *MAT1-2-1* did not vary. Mean values for ENC and GC3 were 52.16 and 0.561 for *MAT1-1-1* and 52.38 and 0.558 for *MAT1-2-1*. Mean CAI values for *Stemphylium MAT1* were also similar, 0.117 for *MAT1-1-1* and 0.113 for *MAT1-2-1*. Though not significantly different, *Stemphylium MAT1* had lower values than *Alternaria* housekeeping genes, indicating lower expression levels.

## Discussion

Almost all known *Alternaria* species are considered to be asexual and only a few *Alternaria* anamorphs have been connected to a teleomorphic stage. A sexual stage for *A. alternata* has never been observed in nature and attempts to produce a sexual stage in the laboratory have failed [8]. Although a sexual stage has never been described for *A. alternata*, this species carries functional genes at the *MAT1* locus [7] and both mating types are routinely recovered from populations of this fungus [T.L. Peever, *unpublished*]. Our codon-site analyses rejected the neutrality hypothesis and indicated that these loci are not evolving neutrally but rather are subject to purifying (negative) selection. Over half the codons (387 aa and 342 aa for *MAT1-1-1* and *MAT1-2-1*, respectively) were under purifying selection. Further, patterns of selection differed between the idiomorphs with *MAT1-1-1* under strong purifying selection with many sites with high posterior probability values and *MAT1-2-1* under weaker purifying selection. Purifying selection might be the result of a cryptic contemporary sexual cycle, a recent sexual past or the involvement of *MAT1* in other critical cellular functions. *Aspergillus fumigatus*, a fungus long thought to be asexual, was recently shown to have a sexual stage [55], which might also be possible for *Alternaria*. Moreover, if the *MAT1* locus in *Alternaria* controls cellular functions in addition to mating, the differences in codon usage between *MAT1-1-1* and *MAT1-2-1* may indicate that genes encoded by each idiomorph have different roles in the cell.

In sexual species, *MAT1-1-1* and *MAT1-2-1* are known to play different roles in the sexual cycle and possibly in other cellular processes [1]. In an elegant study using gene deletions of *MAT1* and *MAT2* in *Aspergillus nidulans*, Paoletti et al. [55] found that mutations affected components of the sexual cycle differentially, particularly the development of thick-walled Hülle cells. The *MAT1-1* alpha box and the *MAT1-2* HMG DNA-binding box motifs are thought to regulate different classes of sex pheromones and their receptors [56] so a differential role in the cell is not unexpected. Another putative role for the *MAT1* locus may be the control of virulence in plant-pathogenic fungi. In a population study, Zhan et al. [57] found that *MAT1-1* strains of the sexual wheat pathogen, *Mycosphaerella graminicola*, were more virulent than *MAT1-2* strains. Limitations in the experimental design did not allow the authors to determine if these associations were due to pleiotropic effects of *MAT1* or other loci tightly linked to *MAT1* [57]. Several studies have suggested that mating-type genes are involved in additional cellular processes such as cell wall maintenance, cellular resistance to DNA damage, and *MAT* pheromones have been shown to induce G proteins which are linked to protein kinase cascades [58]–[60].

Similar to the results presented here, O'Donnell et al. [61] showed that *MAT1* genes in *Fusarium graminearum* were subject to purifying selection, and likewise Rau et al. [62] found amino acid conservation (non-synonymous substitution ratio<synonymous substitution ratio) in *MAT1* genes of *Pyrenophora teres*. Turgeon [4] observed a paucity of silent substitutions in *MAT1* of *C. heterostrophus*, leading to speculation that mating-type genes may be under strong diversifying selection to prevent interspecific mating [63]–[65]. Using likelihood ratio tests of codon site models with and without positive selection, we saw no evidence of overall positive selection at *MAT1* of *Alternaria*, although several positively selected sites within each idiomorph were identified. Several studies have demonstrated heterologous complementation of *MAT*-deficient mutants [7], [14], [66], suggesting that function is retained across genera and this result is not consistent with strong diversifying selection as suggested by Turgeon [4].

Wik et al. [67] found higher levels of positive selection on *MAT1* in heterothallic *Neurospora* species compared to homothallic species. Comparing the results Wik et al. [67] with those presented here for with *Alternaria MAT1*, we find similar numbers of positively selected codons, albeit at different codon sites. Wik et al. [67] found 2, 7 and 1 positively selected codons in *Neurospora* mat-a-1, mat-A-1, and mat A-3, respectively. Similarly, we found that *MAT1-1-1* and *MAT1-2-1* had 5 and 1 positively selected codon sites, respectively. Rare signatures of positive selection scattered within an overall strong signal of purifying selection at *MAT1* in *Alternaria* may not be due to asexuality, but rather rapid divergence in the heterothallic mating system due to adaptive evolution or a lack of selective constraint in the mating type genes [67].

The strength of codon bias can be used to make predictions about expression levels in a gene, where a smaller ENC value is an indicator of the overall codon bias which is then correlated with higher expression levels (higher CAI values) [55]. ENC and CAI values for *Alternaria MAT1* did not follow this trend. *Alternaria MAT1-2-1* showed more codon bias (lower ENC) but also low expression levels (smaller CAI), whereas *MAT1-1-1* had decreased levels of codon bias, but higher expressions levels. The reasons for these differences in codon usage and expression patterns between *Atlernaria MAT1* are unknown. It may signal variation in the type and strength of selective forces. A positive correlation between codon bias and expression level can be attributed to translational efficiency [68]. Codon Adaptation Index (CAI) measures the difference between observed codon frequencies from the null expectation that each amino acid has an equal chance of being encoded by all possible codons, and the difference can be correlated to translational efficiency because as fewer codons are used to encoded amino acids, increasing bias [69]. *Alternaria* housekeeping genes follow this ENC and CAI trend with significant linear correlation (R^2^ = 0.62, *P* = 0.003), whereas for *MAT1* in *Alternaria*, the correlation was weaker and was statistically insignificant (R^2^ = 0.14, *P* = 0.09). We might speculate that lack of correlation between ENC and CAI indicates that translational selection is a weak force in shaping *Alternaria MAT1*. The combined results of GC3, ENC and CAI highlight the possible complexity of selective forces acting on *MAT1* in *Alternaria*.

Our results demonstrating differential codon bias between *MAT1* idiomorphs in *Alternaria* suggest that *MAT1* is conserved. Critical determination of the mating system of any *Alternaria spp.* has not been performed to date but our results may suggest that this genus has a sexual cycle or recent sexual past. The differences detected between *MAT1-1-1* and *MAT1-2-1* may also indicate that the *MAT1* alleles are involved in different biological functions, which may lead to differential fitness between mating types. Future comparisons of *Alternaria MAT1* with *MAT1* from sexual species may help to deduce if the selection and codon bias differences observed in *MAT1-1-1* and *MAT1-2-1* are due to differences in gene function or if they signify divergent histories.

## Supporting Information

Table S1 Isolates used in evolutionary analyses.
(DOCX)
