# Supplementary material for: Contrasting Codon Usage Patterns and Purifying Selection at the Mating Locus in Putatively Asexual Alternaria Fungal Species
Source: PLoS One. 2011 May 19;6(5):e20083. doi: 10.1371/journal.pone.0020083 (PMC3098265; doi:10.1371/journal.pone.0020083)
Supplement: Table S1 — Isolates used in evolutionary analyses. (DOCX) [file pone.0020083.s001.docx]

| Table S1. Isolates used in evolutionary analyses. | | | |
| --- | --- | --- | --- |
| **Species** | **Isolate** | **MAT type** | **Accession #** |
| *Alternaria alternata* | SH-MIL-11s | *MAT1-1-1* | this study |
| *A. alternata* | SH-MIL-22s | *MAT1-1-1* | this study |
| *A. alternata* | SH-MIL-34s | *MAT1-1-1* | this study |
| *A. alternata* | 15A | *MAT1-1-1* | AB009451 |
| *A. citriarbusti* | SH-MIL-38s | *MAT1-1-1* | this study |
| *A. brassicae* | 01-8a | *MAT1-1-1* | this study |
| *A. brassicae* | 01-8b | *MAT1-1-1* | this study |
| *A. brassicicola* | 01-1c-s | *MAT1-1-1* | this study |
| *A. brassicicola* | 01-2a-s | *MAT1-1-1* | this study |
| *A. solani* | IdahoA | *MAT1-1-1* | this study |
| *A. solani* | EGS 44-098 | *MAT1-1-1* | this study |
| *Stemphylium sp.** | P56 | *MAT1-1-1* | AY339852 |
| *Stemphylium sp.** | P107 | *MAT1-1-1* | AAR04443 |
| *Stemphylium loti* | P384 | *MAT1-1-1* | AAR04470 |
| *Stemphylium calilstephi* | P383 | *MAT1-1-1* | AY339863 |
| *Stemphylium sp.* | EGS 49-043 | *MAT1-1-1* | AY339861 |
| *Stemphylium trifolii* | EGS 12-142 | *MAT1-1-1* | AY339856 |
| *Stemphylium sarciniforme* | EGS 38-121 | *MAT1-1-1* | AY339854 |
| *Stemphylium lancipes* | EGS 46-182 | *MAT1-1-1* | AY339853 |
| *Stemphylium solani* | EGS 41-135 | *MAT1-1-1* | AY339855 |
| *Stemphylium sp.* | EGS 44-070 | *MAT1-1-1* | AY339860 |
| *A. alternata* | 0-276 | *MAT1-2-1* | AB009452 |
| *A. alternata* | SH-MIL-13s | *MAT1-2-1* | this study |
| *A. alternata* | SH-MIL-14s | *MAT1-2-1* | this study |
| *A. tangelonis* | SH-MIL-19s | *MAT1-2-1* | this study |
| *A. citriarbusti* | SH-MIL-1s | *MAT1-2-1* | this study |
| *A. brassicicola* | 01-9c-s | *MAT1-2-1* | this study |
| *A. brassicicola* | 01-41a-s | *MAT1-2-1* | this study |
| *A. brassicicola* | 01-23a-s | *MAT1-2-1* | this study |
| *A. solani* | 21ss | *MAT1-2-1* | this study |
| *A. solani* | 39ss | *MAT1-2-1* | this study |
| *S. xanthomonatis* | EGS 17-137 | *MAT1-2-1* | AY340940 |
| *Stemphylium sp.* | EGS 45-031 | *MAT1-2-1* | AY340941 |
| *S. loti* | P385 | *MAT1-2-1* | AY340945 |
| *Stemphylium sp.* | EGS 42-055 | *MAT1-2-1* | AY340942 |
| *S. sarciniforme** | EGS 49-037 | *MAT1-2-1* | AAF87724 |
| *S. sarciniforme** | EGS 49-033 | *MAT1-2-1* | TreeBase*S1383 |
| *A. alternata* | AGA | *G protein alpha subunit* | AB239917 |
| *A. alternata* | AC325 | *endopolygalacturnase* | AB047682 |
| *A. alternata* | unknown | *endoxylanase* | AF176570 |
| *A. alternata* | unknown | *exoglucanse* | AF176571 |
| *A. brassicicola* | Abra43 | *mitogen-activated protein kinase* | AY987486 |
| *A. brassicicola* | ATCC 96866 | *elongation factor alpha 1* | AB04028.1 |
| *A. brassicicola* | ATCC 96866 | *mitogen-activated protein kinase* | AB09565.1 |
| *A. longipes* | Z-26 | *histidine kinase* | ACN62989 |
| *A. alternata* | unknown | *MAP kinase* | GC414506 |
| *A. brassicicola* | Abra40 | *histidine kinase* | AY700098 |
| *A. brassicicola* | Abra43 | *gluathione transferase* | AY987487 |
